# Supplementary material for: Modeling glioblastoma heterogeneity as a dynamic network of cell states
Source: Mol Syst Biol. 2021 Sep 16;17(9):e10105. doi: 10.15252/msb.202010105 (PMC8444284; doi:10.15252/msb.202010105)
Supplement: Supplementary file 5 — Source Data for Figure 3 [file MSB-17-e10105-s001.zip › Figure3A_sourcedata/GSEA_3065/nosigdb_state1.GseaPreranked.1622718409535/RICHARDS_NOWAKOWSKI_SCIENCE_2017_ASTROCYTE_UPREG.html]

Details for gene set RICHARDS\_NOWAKOWSKI\_SCIENCE\_2017\_ASTROCYTE\_UPREG[GSEA]

|  || Dataset | state1 |
| Phenotype | NoPhenotypeAvailable |
| Upregulated in class | na\_neg |
| GeneSet | RICHARDS\_NOWAKOWSKI\_SCIENCE\_2017\_ASTROCYTE\_UPREG |
| Enrichment Score (ES) | -0.63707036 |
| Normalized Enrichment Score (NES) | -2.6727312 |
| Nominal p-value | 0.0 |
| FDR q-value | 0.0 |
| FWER p-Value | 0.0 |
Table: GSEA Results Summary

  

Fig 1: Enrichment plot: RICHARDS\_NOWAKOWSKI\_SCIENCE\_2017\_ASTROCYTE\_UPREG      
 Profile of the Running ES Score & Positions of GeneSet Members on the Rank Ordered List

  

| PROBE | GENE SYMBOL | GENE\_TITLE | RANK IN GENE LIST | RANK METRIC SCORE | RUNNING ES | CORE ENRICHMENT || 1 | LGALS1 |  |  | 4 | 0.828 | 0.0088 | No |
| 2 | PKM |  |  | 14 | 0.676 | 0.0153 | No |
| 3 | S100A13 |  |  | 20 | 0.627 | 0.0218 | No |
| 4 | S100A16 |  |  | 27 | 0.587 | 0.0277 | No |
| 5 | STK17A |  |  | 47 | 0.483 | 0.0310 | No |
| 6 | SPATS2L |  |  | 55 | 0.464 | 0.0354 | No |
| 7 | IGFBP5 |  |  | 88 | 0.385 | 0.0363 | No |
| 8 | ENO1 |  |  | 99 | 0.373 | 0.0394 | No |
| 9 | DBI |  |  | 110 | 0.352 | 0.0422 | No |
| 10 | PSME2 |  |  | 112 | 0.351 | 0.0460 | No |
| 11 | MT2A |  |  | 161 | 0.317 | 0.0445 | No |
| 12 | RGS20 |  |  | 173 | 0.309 | 0.0467 | No |
| 13 | VIM |  |  | 175 | 0.308 | 0.0500 | No |
| 14 | SLC2A1 |  |  | 235 | 0.283 | 0.0469 | No |
| 15 | SLC25A5 |  |  | 254 | 0.276 | 0.0481 | No |
| 16 | TACC1 |  |  | 258 | 0.275 | 0.0508 | No |
| 17 | GNG5 |  |  | 276 | 0.269 | 0.0520 | No |
| 18 | RHOC |  |  | 316 | 0.256 | 0.0507 | No |
| 19 | POLR2L |  |  | 339 | 0.245 | 0.0511 | No |
| 20 | NOG |  |  | 381 | 0.231 | 0.0493 | No |
| 21 | JUN |  |  | 387 | 0.229 | 0.0513 | No |
| 22 | RAB13 |  |  | 417 | 0.223 | 0.0507 | No |
| 23 | SLC7A11 |  |  | 459 | 0.214 | 0.0487 | No |
| 24 | LYPD1 |  |  | 508 | 0.205 | 0.0458 | No |
| 25 | GOLIM4 |  |  | 525 | 0.200 | 0.0464 | No |
| 26 | MPC1 |  |  | 577 | 0.189 | 0.0430 | No |
| 27 | RHOA |  |  | 632 | 0.181 | 0.0393 | No |
| 28 | CAMK2D |  |  | 689 | 0.174 | 0.0353 | No |
| 29 | AXL |  |  | 704 | 0.172 | 0.0357 | No |
| 30 | ANXA5 |  |  | 709 | 0.171 | 0.0372 | No |
| 31 | CNN3 |  |  | 727 | 0.168 | 0.0372 | No |
| 32 | NDUFB3 |  |  | 766 | 0.162 | 0.0350 | No |
| 33 | GCSH |  |  | 771 | 0.162 | 0.0364 | No |
| 34 | ADI1 |  |  | 827 | 0.154 | 0.0322 | No |
| 35 | EFHD2 |  |  | 879 | 0.147 | 0.0284 | No |
| 36 | CXCL14 |  |  | 925 | 0.142 | 0.0252 | No |
| 37 | NTM |  |  | 1002 | 0.134 | 0.0186 | No |
| 38 | MRFAP1 |  |  | 1017 | 0.132 | 0.0186 | No |
| 39 | TRAPPC3 |  |  | 1087 | 0.124 | 0.0126 | No |
| 40 | SPRY2 |  |  | 1171 | 0.115 | 0.0051 | No |
| 41 | ARHGAP12 |  |  | 1174 | 0.115 | 0.0061 | No |
| 42 | PDXK |  |  | 1186 | 0.114 | 0.0062 | No |
| 43 | IRF2 |  |  | 1299 | 0.103 | -0.0046 | No |
| 44 | CD99 |  |  | 1301 | 0.103 | -0.0035 | No |
| 45 | PRDX1 |  |  | 1305 | 0.103 | -0.0027 | No |
| 46 | COL6A1 |  |  | 1317 | 0.102 | -0.0027 | No |
| 47 | DUSP6 |  |  | 1401 | 0.095 | -0.0105 | No |
| 48 | LAP3 |  |  | 1418 | 0.094 | -0.0112 | No |
| 49 | RIN2 |  |  | 1450 | 0.092 | -0.0134 | No |
| 50 | FMNL2 |  |  | 1453 | 0.092 | -0.0126 | No |
| 51 | FABP5 |  |  | 1470 | 0.091 | -0.0133 | No |
| 52 | EGFR |  |  | 1474 | 0.091 | -0.0126 | No |
| 53 | PDLIM5 |  |  | 1528 | 0.087 | -0.0173 | No |
| 54 | CREM |  |  | 1552 | 0.086 | -0.0188 | No |
| 55 | WWC2 |  |  | 1575 | 0.084 | -0.0202 | No |
| 56 | MEST |  |  | 1582 | 0.084 | -0.0199 | No |
| 57 | SNX3 |  |  | 1585 | 0.083 | -0.0192 | No |
| 58 | LIMA1 |  |  | 1678 | 0.077 | -0.0281 | No |
| 59 | PHACTR2 |  |  | 1726 | 0.074 | -0.0323 | No |
| 60 | CHPT1 |  |  | 1808 | 0.069 | -0.0402 | No |
| 61 | NDUFB5 |  |  | 1905 | 0.065 | -0.0497 | No |
| 62 | TEAD1 |  |  | 1913 | 0.065 | -0.0497 | No |
| 63 | FAM120A |  |  | 2038 | 0.058 | -0.0623 | No |
| 64 | SRI |  |  | 2084 | 0.056 | -0.0664 | No |
| 65 | GLUL |  |  | 2130 | 0.054 | -0.0706 | No |
| 66 | GSTM4 |  |  | 2143 | 0.053 | -0.0713 | No |
| 67 | CYR61 |  |  | 2172 | 0.052 | -0.0737 | No |
| 68 | RGCC |  |  | 2214 | 0.051 | -0.0775 | No |
| 69 | LYN |  |  | 2272 | 0.048 | -0.0830 | No |
| 70 | MYO1E |  |  | 2311 | 0.047 | -0.0866 | No |
| 71 | PSMD10 |  |  | 2327 | 0.046 | -0.0877 | No |
| 72 | FAM84B |  |  | 2432 | 0.042 | -0.0983 | No |
| 73 | TIMP1 |  |  | 2497 | 0.040 | -0.1046 | No |
| 74 | LRRC17 |  |  | 2509 | 0.039 | -0.1054 | No |
| 75 | TRIM47 |  |  | 2525 | 0.039 | -0.1065 | No |
| 76 | PHF11 |  |  | 2588 | 0.037 | -0.1127 | No |
| 77 | ZNF639 |  |  | 2678 | 0.034 | -0.1218 | No |
| 78 | ITPK1 |  |  | 2758 | 0.031 | -0.1299 | No |
| 79 | OAT |  |  | 2778 | 0.031 | -0.1316 | No |
| 80 | CHP1 |  |  | 2864 | 0.029 | -0.1403 | No |
| 81 | PPP2R5A |  |  | 2919 | 0.027 | -0.1458 | No |
| 82 | TOMM7 |  |  | 2940 | 0.026 | -0.1476 | No |
| 83 | SCP2 |  |  | 3047 | 0.024 | -0.1586 | No |
| 84 | RGMA |  |  | 3071 | 0.023 | -0.1608 | No |
| 85 | GNG12 |  |  | 3088 | 0.023 | -0.1623 | No |
| 86 | TLK1 |  |  | 3089 | 0.023 | -0.1620 | No |
| 87 | GULP1 |  |  | 3139 | 0.022 | -0.1670 | No |
| 88 | RASSF2 |  |  | 3195 | 0.020 | -0.1726 | No |
| 89 | SNTA1 |  |  | 3196 | 0.020 | -0.1724 | No |
| 90 | GLUD1 |  |  | 3290 | 0.018 | -0.1821 | No |
| 91 | MCC |  |  | 3348 | 0.017 | -0.1880 | No |
| 92 | DST |  |  | 3373 | 0.017 | -0.1903 | No |
| 93 | OGFRL1 |  |  | 3377 | 0.016 | -0.1905 | No |
| 94 | CDCA7L |  |  | 3390 | 0.016 | -0.1916 | No |
| 95 | MAST4 |  |  | 3414 | 0.016 | -0.1938 | No |
| 96 | EEPD1 |  |  | 3518 | 0.013 | -0.2046 | No |
| 97 | ATF7 |  |  | 3523 | 0.013 | -0.2049 | No |
| 98 | MLEC |  |  | 3567 | 0.012 | -0.2094 | No |
| 99 | CXXC5 |  |  | 3632 | 0.011 | -0.2161 | No |
| 100 | AHCYL1 |  |  | 3686 | 0.010 | -0.2216 | No |
| 101 | CHKA |  |  | 3687 | 0.010 | -0.2215 | No |
| 102 | SLC25A18 |  |  | 3696 | 0.010 | -0.2222 | No |
| 103 | THG1L |  |  | 3700 | 0.010 | -0.2224 | No |
| 104 | SSR3 |  |  | 3768 | 0.009 | -0.2295 | No |
| 105 | SPRED2 |  |  | 3780 | 0.008 | -0.2305 | No |
| 106 | SUCLG2 |  |  | 3841 | 0.007 | -0.2368 | No |
| 107 | NDUFA4 |  |  | 3842 | 0.007 | -0.2368 | No |
| 108 | ETV5 |  |  | 3894 | 0.006 | -0.2421 | No |
| 109 | APOL2 |  |  | 3946 | 0.005 | -0.2475 | No |
| 110 | LITAF |  |  | 4039 | 0.003 | -0.2572 | No |
| 111 | SMC5 |  |  | 4048 | 0.003 | -0.2581 | No |
| 112 | NEK1 |  |  | 4071 | 0.003 | -0.2604 | No |
| 113 | VEGFB |  |  | 4079 | 0.003 | -0.2611 | No |
| 114 | DPF3 |  |  | 4225 | 0.000 | -0.2765 | No |
| 115 | PSAT1 |  |  | 4318 | -0.001 | -0.2863 | No |
| 116 | PRTFDC1 |  |  | 4344 | -0.002 | -0.2889 | No |
| 117 | SNAP23 |  |  | 4402 | -0.003 | -0.2950 | No |
| 118 | KAT2A |  |  | 4427 | -0.003 | -0.2975 | No |
| 119 | PEX2 |  |  | 4433 | -0.003 | -0.2980 | No |
| 120 | SASH1 |  |  | 4498 | -0.004 | -0.3048 | No |
| 121 | BBX |  |  | 4535 | -0.005 | -0.3085 | No |
| 122 | GLDC |  |  | 4543 | -0.005 | -0.3092 | No |
| 123 | BMP7 |  |  | 4562 | -0.006 | -0.3111 | No |
| 124 | SEL1L3 |  |  | 4651 | -0.007 | -0.3204 | No |
| 125 | PARD3 |  |  | 4658 | -0.007 | -0.3209 | No |
| 126 | TCF7L2 |  |  | 4707 | -0.008 | -0.3259 | No |
| 127 | LDHB |  |  | 4712 | -0.008 | -0.3263 | No |
| 128 | ABI1 |  |  | 4714 | -0.008 | -0.3263 | No |
| 129 | ZIC1 |  |  | 4766 | -0.008 | -0.3316 | No |
| 130 | CRYL1 |  |  | 4799 | -0.009 | -0.3350 | No |
| 131 | CLDND1 |  |  | 4823 | -0.009 | -0.3373 | No |
| 132 | SWAP70 |  |  | 4882 | -0.011 | -0.3434 | No |
| 133 | RHOJ |  |  | 5068 | -0.014 | -0.3629 | No |
| 134 | SCCPDH |  |  | 5110 | -0.014 | -0.3671 | No |
| 135 | PHGDH |  |  | 5207 | -0.016 | -0.3772 | No |
| 136 | CERS6 |  |  | 5229 | -0.016 | -0.3792 | No |
| 137 | ARHGAP5 |  |  | 5327 | -0.017 | -0.3893 | No |
| 138 | MTMR10 |  |  | 5460 | -0.019 | -0.4032 | No |
| 139 | RNPC3 |  |  | 5601 | -0.022 | -0.4178 | No |
| 140 | CNDP2 |  |  | 5710 | -0.024 | -0.4291 | No |
| 141 | BEND7 |  |  | 5791 | -0.025 | -0.4373 | No |
| 142 | EFCAB2 |  |  | 5873 | -0.027 | -0.4456 | No |
| 143 | MCCC2 |  |  | 5900 | -0.027 | -0.4481 | No |
| 144 | SFXN5 |  |  | 5908 | -0.028 | -0.4485 | No |
| 145 | PCCB |  |  | 5924 | -0.028 | -0.4498 | No |
| 146 | NEK6 |  |  | 5956 | -0.028 | -0.4528 | No |
| 147 | ROBO3 |  |  | 5970 | -0.029 | -0.4539 | No |
| 148 | USP46 |  |  | 5973 | -0.029 | -0.4538 | No |
| 149 | DTNA |  |  | 6001 | -0.029 | -0.4563 | No |
| 150 | FUT11 |  |  | 6068 | -0.030 | -0.4630 | No |
| 151 | SLC25A53 |  |  | 6082 | -0.030 | -0.4641 | No |
| 152 | HNRNPA2B1 |  |  | 6142 | -0.032 | -0.4700 | No |
| 153 | ABCD3 |  |  | 6145 | -0.032 | -0.4698 | No |
| 154 | IDI1 |  |  | 6154 | -0.032 | -0.4703 | No |
| 155 | MTSS1L |  |  | 6165 | -0.032 | -0.4711 | No |
| 156 | SRPK2 |  |  | 6188 | -0.033 | -0.4730 | No |
| 157 | MCFD2 |  |  | 6221 | -0.033 | -0.4761 | No |
| 158 | IMPACT |  |  | 6315 | -0.035 | -0.4856 | No |
| 159 | TGIF1 |  |  | 6360 | -0.036 | -0.4899 | No |
| 160 | KCNJ16 |  |  | 6368 | -0.036 | -0.4902 | No |
| 161 | METTL8 |  |  | 6396 | -0.036 | -0.4927 | No |
| 162 | ZNF521 |  |  | 6445 | -0.037 | -0.4974 | No |
| 163 | MT1X |  |  | 6479 | -0.038 | -0.5005 | No |
| 164 | WDR41 |  |  | 6500 | -0.039 | -0.5022 | No |
| 165 | GAS1 |  |  | 6510 | -0.039 | -0.5027 | No |
| 166 | FERMT2 |  |  | 6579 | -0.040 | -0.5095 | No |
| 167 | DPP8 |  |  | 6645 | -0.042 | -0.5159 | No |
| 168 | TOB2 |  |  | 6650 | -0.042 | -0.5159 | No |
| 169 | PRNP |  |  | 6661 | -0.042 | -0.5165 | No |
| 170 | RHBDD2 |  |  | 6682 | -0.043 | -0.5182 | No |
| 171 | ID1 |  |  | 6749 | -0.044 | -0.5247 | No |
| 172 | RASSF4 |  |  | 6781 | -0.045 | -0.5275 | No |
| 173 | NBPF14 |  |  | 6792 | -0.045 | -0.5281 | No |
| 174 | VPS36 |  |  | 6821 | -0.045 | -0.5305 | No |
| 175 | DPM3 |  |  | 6825 | -0.045 | -0.5304 | No |
| 176 | PEA15 |  |  | 6830 | -0.046 | -0.5303 | No |
| 177 | CAMK2G |  |  | 6872 | -0.046 | -0.5341 | No |
| 178 | SLC48A1 |  |  | 6888 | -0.047 | -0.5352 | No |
| 179 | EFCAB14 |  |  | 6909 | -0.048 | -0.5368 | No |
| 180 | SIRPA |  |  | 6912 | -0.048 | -0.5365 | No |
| 181 | SSPN |  |  | 6940 | -0.048 | -0.5388 | No |
| 182 | DDAH1 |  |  | 6943 | -0.049 | -0.5385 | No |
| 183 | CLNS1A |  |  | 7008 | -0.050 | -0.5447 | No |
| 184 | SNX5 |  |  | 7025 | -0.051 | -0.5459 | No |
| 185 | BMPR1A |  |  | 7093 | -0.053 | -0.5524 | No |
| 186 | SRGAP2B |  |  | 7119 | -0.054 | -0.5545 | No |
| 187 | ZC3HAV1 |  |  | 7178 | -0.055 | -0.5601 | No |
| 188 | PTPRD |  |  | 7218 | -0.056 | -0.5636 | No |
| 189 | ERAP1 |  |  | 7219 | -0.056 | -0.5630 | No |
| 190 | RAB31 |  |  | 7256 | -0.057 | -0.5662 | No |
| 191 | PBXIP1 |  |  | 7302 | -0.058 | -0.5703 | No |
| 192 | IQSEC1 |  |  | 7311 | -0.058 | -0.5705 | No |
| 193 | PAQR8 |  |  | 7316 | -0.058 | -0.5703 | No |
| 194 | ALDH6A1 |  |  | 7321 | -0.059 | -0.5701 | No |
| 195 | ITGA6 |  |  | 7339 | -0.059 | -0.5712 | No |
| 196 | IVD |  |  | 7376 | -0.060 | -0.5744 | No |
| 197 | PLEKHG1 |  |  | 7408 | -0.061 | -0.5770 | No |
| 198 | JAG1 |  |  | 7426 | -0.061 | -0.5781 | No |
| 199 | SERPINH1 |  |  | 7462 | -0.062 | -0.5812 | No |
| 200 | ABCA3 |  |  | 7467 | -0.062 | -0.5809 | No |
| 201 | GJA1 |  |  | 7471 | -0.062 | -0.5805 | No |
| 202 | COG2 |  |  | 7488 | -0.063 | -0.5815 | No |
| 203 | DOCK1 |  |  | 7505 | -0.064 | -0.5825 | No |
| 204 | CCDC80 |  |  | 7525 | -0.064 | -0.5838 | No |
| 205 | STK3 |  |  | 7560 | -0.065 | -0.5867 | No |
| 206 | IFT88 |  |  | 7573 | -0.066 | -0.5873 | No |
| 207 | NADK2 |  |  | 7596 | -0.066 | -0.5889 | No |
| 208 | STAT3 |  |  | 7597 | -0.066 | -0.5882 | No |
| 209 | SLC18B1 |  |  | 7663 | -0.068 | -0.5943 | No |
| 210 | ACOX1 |  |  | 7691 | -0.069 | -0.5964 | No |
| 211 | DGKH |  |  | 7702 | -0.070 | -0.5967 | No |
| 212 | GRIA4 |  |  | 7708 | -0.070 | -0.5965 | No |
| 213 | SPRED1 |  |  | 7743 | -0.071 | -0.5993 | No |
| 214 | PCDH17 |  |  | 7749 | -0.072 | -0.5990 | No |
| 215 | MAPK1 |  |  | 7779 | -0.072 | -0.6013 | No |
| 216 | MRPS5 |  |  | 7783 | -0.073 | -0.6008 | No |
| 217 | MEIS1 |  |  | 7794 | -0.073 | -0.6011 | No |
| 218 | ABLIM1 |  |  | 7806 | -0.074 | -0.6014 | No |
| 219 | HOPX |  |  | 7850 | -0.075 | -0.6052 | No |
| 220 | DNER |  |  | 7852 | -0.075 | -0.6044 | No |
| 221 | BBS2 |  |  | 7875 | -0.076 | -0.6059 | No |
| 222 | ADAMTS6 |  |  | 7912 | -0.078 | -0.6089 | No |
| 223 | HEATR5A |  |  | 7916 | -0.078 | -0.6083 | No |
| 224 | TMEM9B |  |  | 7929 | -0.078 | -0.6087 | No |
| 225 | IFI27L1 |  |  | 7968 | -0.080 | -0.6119 | No |
| 226 | SEPT8 |  |  | 7971 | -0.080 | -0.6112 | No |
| 227 | PARP14 |  |  | 7988 | -0.081 | -0.6120 | No |
| 228 | FJX1 |  |  | 8037 | -0.083 | -0.6162 | No |
| 229 | PURB |  |  | 8047 | -0.083 | -0.6162 | No |
| 230 | GNG7 |  |  | 8050 | -0.083 | -0.6155 | No |
| 231 | PPIB |  |  | 8099 | -0.085 | -0.6197 | No |
| 232 | AGPAT3 |  |  | 8118 | -0.086 | -0.6207 | No |
| 233 | ZNF236 |  |  | 8163 | -0.088 | -0.6244 | No |
| 234 | ENHO |  |  | 8176 | -0.089 | -0.6247 | No |
| 235 | LRP4 |  |  | 8181 | -0.089 | -0.6241 | No |
| 236 | NDRG4 |  |  | 8182 | -0.089 | -0.6231 | No |
| 237 | SC5D |  |  | 8206 | -0.090 | -0.6246 | No |
| 238 | SLC35A5 |  |  | 8207 | -0.090 | -0.6236 | No |
| 239 | SLC38A3 |  |  | 8217 | -0.090 | -0.6235 | No |
| 240 | SAMD8 |  |  | 8220 | -0.090 | -0.6227 | No |
| 241 | HSDL2 |  |  | 8225 | -0.090 | -0.6221 | No |
| 242 | LPP |  |  | 8255 | -0.092 | -0.6242 | No |
| 243 | PDLIM3 |  |  | 8278 | -0.093 | -0.6255 | No |
| 244 | NCKAP5 |  |  | 8280 | -0.094 | -0.6246 | No |
| 245 | UGP2 |  |  | 8287 | -0.094 | -0.6242 | No |
| 246 | CASC4 |  |  | 8309 | -0.095 | -0.6254 | No |
| 247 | ADCY2 |  |  | 8311 | -0.095 | -0.6244 | No |
| 248 | GABBR1 |  |  | 8312 | -0.095 | -0.6233 | No |
| 249 | PNPT1 |  |  | 8313 | -0.095 | -0.6223 | No |
| 250 | IFI6 |  |  | 8316 | -0.095 | -0.6214 | No |
| 251 | ADD3 |  |  | 8338 | -0.096 | -0.6226 | No |
| 252 | LARS |  |  | 8345 | -0.096 | -0.6222 | No |
| 253 | TNFRSF19 |  |  | 8355 | -0.097 | -0.6221 | No |
| 254 | ID2 |  |  | 8360 | -0.097 | -0.6214 | No |
| 255 | C3orf70 |  |  | 8375 | -0.098 | -0.6218 | No |
| 256 | EPB41L2 |  |  | 8402 | -0.099 | -0.6235 | No |
| 257 | MED13 |  |  | 8421 | -0.100 | -0.6243 | No |
| 258 | PSENEN |  |  | 8463 | -0.103 | -0.6275 | No |
| 259 | ALDH7A1 |  |  | 8467 | -0.103 | -0.6267 | No |
| 260 | TGFB2 |  |  | 8497 | -0.105 | -0.6286 | No |
| 261 | TAPBP |  |  | 8522 | -0.106 | -0.6300 | No |
| 262 | ZDHHC21 |  |  | 8530 | -0.107 | -0.6295 | No |
| 263 | SYPL1 |  |  | 8546 | -0.108 | -0.6299 | No |
| 264 | RCN1 |  |  | 8553 | -0.108 | -0.6294 | No |
| 265 | UBL3 |  |  | 8573 | -0.110 | -0.6302 | No |
| 266 | SESN3 |  |  | 8632 | -0.113 | -0.6351 | No |
| 267 | CA2 |  |  | 8636 | -0.113 | -0.6341 | No |
| 268 | PARP9 |  |  | 8650 | -0.114 | -0.6342 | No |
| 269 | DAD1 |  |  | 8655 | -0.114 | -0.6334 | No |
| 270 | COL11A1 |  |  | 8666 | -0.115 | -0.6332 | No |
| 271 | FAM3C |  |  | 8687 | -0.116 | -0.6340 | No |
| 272 | HS2ST1 |  |  | 8696 | -0.117 | -0.6336 | No |
| 273 | MLLT6 |  |  | 8712 | -0.118 | -0.6338 | No |
| 274 | HADHB |  |  | 8718 | -0.119 | -0.6331 | No |
| 275 | AIF1L |  |  | 8722 | -0.119 | -0.6320 | No |
| 276 | SERPINE2 |  |  | 8736 | -0.120 | -0.6321 | No |
| 277 | MAP3K1 |  |  | 8750 | -0.121 | -0.6321 | No |
| 278 | KAT2B |  |  | 8754 | -0.122 | -0.6311 | No |
| 279 | PTCH1 |  |  | 8777 | -0.123 | -0.6321 | No |
| 280 | MYO6 |  |  | 8788 | -0.124 | -0.6317 | No |
| 281 | ZFP36L1 |  |  | 8789 | -0.124 | -0.6304 | No |
| 282 | TRIB2 |  |  | 8853 | -0.129 | -0.6356 | Yes |
| 283 | C2orf72 |  |  | 8855 | -0.129 | -0.6343 | Yes |
| 284 | ID4 |  |  | 8856 | -0.129 | -0.6329 | Yes |
| 285 | ZNF621 |  |  | 8863 | -0.130 | -0.6321 | Yes |
| 286 | ARHGAP31 |  |  | 8865 | -0.130 | -0.6307 | Yes |
| 287 | TMEM170A |  |  | 8873 | -0.131 | -0.6300 | Yes |
| 288 | NACC2 |  |  | 8875 | -0.131 | -0.6287 | Yes |
| 289 | ZCCHC24 |  |  | 8878 | -0.132 | -0.6274 | Yes |
| 290 | CSGALNACT1 |  |  | 8884 | -0.132 | -0.6265 | Yes |
| 291 | ARAP2 |  |  | 8907 | -0.134 | -0.6273 | Yes |
| 292 | BDH2 |  |  | 8927 | -0.136 | -0.6278 | Yes |
| 293 | NTRK3 |  |  | 8930 | -0.137 | -0.6265 | Yes |
| 294 | PHYHIPL |  |  | 8937 | -0.137 | -0.6256 | Yes |
| 295 | ZMAT1 |  |  | 8947 | -0.138 | -0.6250 | Yes |
| 296 | MT3 |  |  | 8954 | -0.139 | -0.6241 | Yes |
| 297 | FAM19A5 |  |  | 8956 | -0.139 | -0.6227 | Yes |
| 298 | UFL1 |  |  | 8970 | -0.140 | -0.6225 | Yes |
| 299 | ZFYVE16 |  |  | 8992 | -0.142 | -0.6232 | Yes |
| 300 | GATM |  |  | 9049 | -0.147 | -0.6275 | Yes |
| 301 | LRRN1 |  |  | 9054 | -0.147 | -0.6263 | Yes |
| 302 | PTAR1 |  |  | 9056 | -0.148 | -0.6247 | Yes |
| 303 | COMT |  |  | 9058 | -0.148 | -0.6232 | Yes |
| 304 | IFNAR1 |  |  | 9068 | -0.149 | -0.6225 | Yes |
| 305 | LPIN2 |  |  | 9075 | -0.150 | -0.6215 | Yes |
| 306 | FADS2 |  |  | 9078 | -0.150 | -0.6200 | Yes |
| 307 | ST3GAL5 |  |  | 9085 | -0.151 | -0.6190 | Yes |
| 308 | HIBCH |  |  | 9087 | -0.151 | -0.6174 | Yes |
| 309 | SPPL2A |  |  | 9100 | -0.152 | -0.6170 | Yes |
| 310 | CD63 |  |  | 9126 | -0.154 | -0.6179 | Yes |
| 311 | NLGN1 |  |  | 9131 | -0.155 | -0.6166 | Yes |
| 312 | ARHGEF6 |  |  | 9135 | -0.156 | -0.6152 | Yes |
| 313 | CADM2 |  |  | 9141 | -0.157 | -0.6140 | Yes |
| 314 | LHX2 |  |  | 9142 | -0.157 | -0.6123 | Yes |
| 315 | DNASE2 |  |  | 9145 | -0.157 | -0.6107 | Yes |
| 316 | CPE |  |  | 9147 | -0.157 | -0.6091 | Yes |
| 317 | ZFP36L2 |  |  | 9162 | -0.160 | -0.6088 | Yes |
| 318 | ATP9B |  |  | 9164 | -0.161 | -0.6071 | Yes |
| 319 | MTRNR2L8 |  |  | 9168 | -0.161 | -0.6056 | Yes |
| 320 | PLOD2 |  |  | 9169 | -0.161 | -0.6038 | Yes |
| 321 | ZMYM2 |  |  | 9171 | -0.162 | -0.6022 | Yes |
| 322 | LRP1B |  |  | 9174 | -0.162 | -0.6006 | Yes |
| 323 | NPAS3 |  |  | 9181 | -0.163 | -0.5994 | Yes |
| 324 | OPHN1 |  |  | 9189 | -0.164 | -0.5983 | Yes |
| 325 | GPC6 |  |  | 9204 | -0.166 | -0.5980 | Yes |
| 326 | IGFBP2 |  |  | 9209 | -0.167 | -0.5965 | Yes |
| 327 | HEY1 |  |  | 9215 | -0.168 | -0.5952 | Yes |
| 328 | LIFR |  |  | 9216 | -0.168 | -0.5933 | Yes |
| 329 | VEGFA |  |  | 9220 | -0.168 | -0.5918 | Yes |
| 330 | RNF180 |  |  | 9225 | -0.169 | -0.5903 | Yes |
| 331 | KANK1 |  |  | 9229 | -0.169 | -0.5887 | Yes |
| 332 | AASS |  |  | 9237 | -0.171 | -0.5876 | Yes |
| 333 | SEPT2 |  |  | 9246 | -0.172 | -0.5865 | Yes |
| 334 | LRIG1 |  |  | 9256 | -0.173 | -0.5856 | Yes |
| 335 | NOTCH2 |  |  | 9279 | -0.177 | -0.5859 | Yes |
| 336 | HES1 |  |  | 9280 | -0.177 | -0.5840 | Yes |
| 337 | ARHGEF26 |  |  | 9286 | -0.178 | -0.5825 | Yes |
| 338 | BAALC |  |  | 9292 | -0.179 | -0.5811 | Yes |
| 339 | CTNNB1 |  |  | 9294 | -0.179 | -0.5792 | Yes |
| 340 | ZMAT3 |  |  | 9300 | -0.181 | -0.5777 | Yes |
| 341 | FAT1 |  |  | 9301 | -0.181 | -0.5757 | Yes |
| 342 | TNK2 |  |  | 9333 | -0.187 | -0.5769 | Yes |
| 343 | NRBP2 |  |  | 9350 | -0.190 | -0.5765 | Yes |
| 344 | TAOK3 |  |  | 9351 | -0.190 | -0.5744 | Yes |
| 345 | MEGF10 |  |  | 9353 | -0.190 | -0.5724 | Yes |
| 346 | HSP90B1 |  |  | 9365 | -0.193 | -0.5714 | Yes |
| 347 | PTPRG |  |  | 9370 | -0.194 | -0.5697 | Yes |
| 348 | NLGN4X |  |  | 9377 | -0.196 | -0.5681 | Yes |
| 349 | TRPS1 |  |  | 9385 | -0.198 | -0.5667 | Yes |
| 350 | MSMO1 |  |  | 9391 | -0.199 | -0.5650 | Yes |
| 351 | PMP22 |  |  | 9392 | -0.199 | -0.5628 | Yes |
| 352 | FHL1 |  |  | 9398 | -0.200 | -0.5611 | Yes |
| 353 | RFTN2 |  |  | 9402 | -0.200 | -0.5592 | Yes |
| 354 | TFPI |  |  | 9427 | -0.206 | -0.5594 | Yes |
| 355 | CD81 |  |  | 9434 | -0.208 | -0.5578 | Yes |
| 356 | HSD17B12 |  |  | 9436 | -0.208 | -0.5556 | Yes |
| 357 | ATP1B2 |  |  | 9441 | -0.210 | -0.5537 | Yes |
| 358 | SEMA6D |  |  | 9442 | -0.210 | -0.5513 | Yes |
| 359 | LGALS3BP |  |  | 9447 | -0.212 | -0.5494 | Yes |
| 360 | APCDD1 |  |  | 9452 | -0.214 | -0.5474 | Yes |
| 361 | GPRC5B |  |  | 9454 | -0.214 | -0.5452 | Yes |
| 362 | FKBP10 |  |  | 9455 | -0.215 | -0.5428 | Yes |
| 363 | SOX8 |  |  | 9456 | -0.215 | -0.5404 | Yes |
| 364 | PHLPP1 |  |  | 9463 | -0.217 | -0.5386 | Yes |
| 365 | SPECC1 |  |  | 9470 | -0.219 | -0.5368 | Yes |
| 366 | PIK3R1 |  |  | 9474 | -0.220 | -0.5347 | Yes |
| 367 | ITM2B |  |  | 9486 | -0.223 | -0.5334 | Yes |
| 368 | ZEB1 |  |  | 9492 | -0.224 | -0.5314 | Yes |
| 369 | NOTCH3 |  |  | 9501 | -0.226 | -0.5298 | Yes |
| 370 | CLSTN1 |  |  | 9504 | -0.227 | -0.5275 | Yes |
| 371 | SLC16A1 |  |  | 9509 | -0.227 | -0.5254 | Yes |
| 372 | TRIM9 |  |  | 9512 | -0.229 | -0.5230 | Yes |
| 373 | DAG1 |  |  | 9514 | -0.229 | -0.5206 | Yes |
| 374 | HTRA1 |  |  | 9515 | -0.229 | -0.5180 | Yes |
| 375 | IL6ST |  |  | 9519 | -0.231 | -0.5158 | Yes |
| 376 | PDE4B |  |  | 9522 | -0.231 | -0.5134 | Yes |
| 377 | NLGN3 |  |  | 9554 | -0.241 | -0.5141 | Yes |
| 378 | HEPACAM |  |  | 9555 | -0.242 | -0.5114 | Yes |
| 379 | ITPR2 |  |  | 9560 | -0.244 | -0.5091 | Yes |
| 380 | PTPN13 |  |  | 9562 | -0.245 | -0.5064 | Yes |
| 381 | FOS |  |  | 9566 | -0.247 | -0.5040 | Yes |
| 382 | LDLRAD3 |  |  | 9567 | -0.247 | -0.5013 | Yes |
| 383 | FYN |  |  | 9575 | -0.250 | -0.4992 | Yes |
| 384 | MAGI1 |  |  | 9576 | -0.250 | -0.4965 | Yes |
| 385 | APC |  |  | 9577 | -0.251 | -0.4937 | Yes |
| 386 | TTC14 |  |  | 9578 | -0.252 | -0.4909 | Yes |
| 387 | CST3 |  |  | 9581 | -0.254 | -0.4883 | Yes |
| 388 | PCDHGC3 |  |  | 9588 | -0.255 | -0.4861 | Yes |
| 389 | TJP1 |  |  | 9592 | -0.257 | -0.4835 | Yes |
| 390 | CTNND2 |  |  | 9595 | -0.257 | -0.4809 | Yes |
| 391 | TNC |  |  | 9605 | -0.262 | -0.4789 | Yes |
| 392 | DPP6 |  |  | 9606 | -0.262 | -0.4760 | Yes |
| 393 | JAM2 |  |  | 9612 | -0.263 | -0.4736 | Yes |
| 394 | TMEM132B |  |  | 9613 | -0.264 | -0.4707 | Yes |
| 395 | FGFR3 |  |  | 9621 | -0.266 | -0.4685 | Yes |
| 396 | PTPRA |  |  | 9624 | -0.268 | -0.4657 | Yes |
| 397 | PNISR |  |  | 9627 | -0.272 | -0.4629 | Yes |
| 398 | SEZ6L |  |  | 9628 | -0.272 | -0.4599 | Yes |
| 399 | TIMP2 |  |  | 9634 | -0.274 | -0.4573 | Yes |
| 400 | TXNIP |  |  | 9639 | -0.276 | -0.4547 | Yes |
| 401 | MAML2 |  |  | 9642 | -0.277 | -0.4518 | Yes |
| 402 | ABAT |  |  | 9646 | -0.278 | -0.4491 | Yes |
| 403 | ITGAV |  |  | 9650 | -0.282 | -0.4462 | Yes |
| 404 | RFX4 |  |  | 9655 | -0.285 | -0.4435 | Yes |
| 405 | SPATA6 |  |  | 9657 | -0.286 | -0.4404 | Yes |
| 406 | LRRN3 |  |  | 9659 | -0.286 | -0.4373 | Yes |
| 407 | HIPK2 |  |  | 9660 | -0.286 | -0.4342 | Yes |
| 408 | MFGE8 |  |  | 9665 | -0.291 | -0.4313 | Yes |
| 409 | PLEKHH2 |  |  | 9666 | -0.291 | -0.4281 | Yes |
| 410 | SOX2 |  |  | 9680 | -0.298 | -0.4262 | Yes |
| 411 | LRRC4C |  |  | 9682 | -0.299 | -0.4230 | Yes |
| 412 | BCHE |  |  | 9684 | -0.300 | -0.4197 | Yes |
| 413 | SEMA6A |  |  | 9690 | -0.304 | -0.4169 | Yes |
| 414 | NTRK2 |  |  | 9692 | -0.307 | -0.4136 | Yes |
| 415 | TMEM106B |  |  | 9693 | -0.308 | -0.4101 | Yes |
| 416 | RNF13 |  |  | 9703 | -0.314 | -0.4076 | Yes |
| 417 | ILDR2 |  |  | 9708 | -0.323 | -0.4044 | Yes |
| 418 | TSPAN3 |  |  | 9713 | -0.326 | -0.4012 | Yes |
| 419 | DSEL |  |  | 9714 | -0.327 | -0.3976 | Yes |
| 420 | SLC35F1 |  |  | 9715 | -0.327 | -0.3940 | Yes |
| 421 | TMBIM6 |  |  | 9716 | -0.328 | -0.3903 | Yes |
| 422 | DCLK2 |  |  | 9717 | -0.328 | -0.3867 | Yes |
| 423 | HMGCS1 |  |  | 9718 | -0.329 | -0.3830 | Yes |
| 424 | CSPG5 |  |  | 9721 | -0.330 | -0.3795 | Yes |
| 425 | MMP16 |  |  | 9724 | -0.335 | -0.3760 | Yes |
| 426 | MYO10 |  |  | 9725 | -0.335 | -0.3723 | Yes |
| 427 | MLC1 |  |  | 9728 | -0.338 | -0.3688 | Yes |
| 428 | LAMB2 |  |  | 9734 | -0.344 | -0.3655 | Yes |
| 429 | IDH1 |  |  | 9737 | -0.345 | -0.3618 | Yes |
| 430 | PSAP |  |  | 9742 | -0.351 | -0.3584 | Yes |
| 431 | ZBTB20 |  |  | 9743 | -0.352 | -0.3544 | Yes |
| 432 | PCDH9 |  |  | 9746 | -0.353 | -0.3507 | Yes |
| 433 | SLC4A4 |  |  | 9749 | -0.355 | -0.3470 | Yes |
| 434 | TMED10 |  |  | 9750 | -0.356 | -0.3430 | Yes |
| 435 | SOX9 |  |  | 9754 | -0.358 | -0.3394 | Yes |
| 436 | SDC3 |  |  | 9756 | -0.360 | -0.3355 | Yes |
| 437 | ITM2C |  |  | 9758 | -0.363 | -0.3315 | Yes |
| 438 | OLFM2 |  |  | 9760 | -0.365 | -0.3276 | Yes |
| 439 | SCD |  |  | 9762 | -0.370 | -0.3236 | Yes |
| 440 | ETV1 |  |  | 9764 | -0.371 | -0.3196 | Yes |
| 441 | LRP1 |  |  | 9771 | -0.381 | -0.3160 | Yes |
| 442 | CADM4 |  |  | 9775 | -0.385 | -0.3120 | Yes |
| 443 | OLIG2 |  |  | 9776 | -0.386 | -0.3077 | Yes |
| 444 | NOTCH1 |  |  | 9780 | -0.391 | -0.3037 | Yes |
| 445 | SYT11 |  |  | 9785 | -0.399 | -0.2997 | Yes |
| 446 | RHOBTB3 |  |  | 9789 | -0.406 | -0.2955 | Yes |
| 447 | B2M |  |  | 9791 | -0.408 | -0.2910 | Yes |
| 448 | ITGB8 |  |  | 9794 | -0.418 | -0.2866 | Yes |
| 449 | CLU |  |  | 9795 | -0.418 | -0.2819 | Yes |
| 450 | SPARCL1 |  |  | 9798 | -0.423 | -0.2774 | Yes |
| 451 | QKI |  |  | 9803 | -0.430 | -0.2731 | Yes |
| 452 | CANX |  |  | 9804 | -0.431 | -0.2683 | Yes |
| 453 | PDGFRA |  |  | 9806 | -0.440 | -0.2635 | Yes |
| 454 | SAT1 |  |  | 9807 | -0.445 | -0.2585 | Yes |
| 455 | WSCD1 |  |  | 9808 | -0.448 | -0.2536 | Yes |
| 456 | ABHD2 |  |  | 9812 | -0.476 | -0.2486 | Yes |
| 457 | C1orf61 |  |  | 9813 | -0.479 | -0.2433 | Yes |
| 458 | LSAMP |  |  | 9814 | -0.481 | -0.2379 | Yes |
| 459 | ALCAM |  |  | 9818 | -0.493 | -0.2327 | Yes |
| 460 | GPM6A |  |  | 9820 | -0.499 | -0.2273 | Yes |
| 461 | SEPT7 |  |  | 9821 | -0.500 | -0.2217 | Yes |
| 462 | S100B |  |  | 9822 | -0.507 | -0.2161 | Yes |
| 463 | SLC1A3 |  |  | 9823 | -0.509 | -0.2104 | Yes |
| 464 | EDNRB |  |  | 9824 | -0.509 | -0.2048 | Yes |
| 465 | FABP7 |  |  | 9825 | -0.513 | -0.1990 | Yes |
| 466 | DDR1 |  |  | 9829 | -0.524 | -0.1935 | Yes |
| 467 | CD9 |  |  | 9832 | -0.544 | -0.1877 | Yes |
| 468 | PON2 |  |  | 9835 | -0.554 | -0.1817 | Yes |
| 469 | WSB1 |  |  | 9836 | -0.554 | -0.1756 | Yes |
| 470 | SCD5 |  |  | 9838 | -0.576 | -0.1693 | Yes |
| 471 | LAPTM4B |  |  | 9840 | -0.579 | -0.1629 | Yes |
| 472 | OLIG1 |  |  | 9845 | -0.637 | -0.1563 | Yes |
| 473 | FGFBP3 |  |  | 9850 | -0.651 | -0.1495 | Yes |
| 474 | FAM181B |  |  | 9854 | -0.697 | -0.1420 | Yes |
| 475 | PRCP |  |  | 9855 | -0.712 | -0.1341 | Yes |
| 476 | NOVA1 |  |  | 9858 | -0.741 | -0.1261 | Yes |
| 477 | SEMA5A |  |  | 9860 | -0.749 | -0.1179 | Yes |
| 478 | NEAT1 |  |  | 9866 | -0.816 | -0.1093 | Yes |
| 479 | MALAT1 |  |  | 9868 | -0.912 | -0.0993 | Yes |
| 480 | PTPRZ1 |  |  | 9869 | -0.918 | -0.0891 | Yes |
| 481 | PMP2 |  |  | 9871 | -0.982 | -0.0783 | Yes |
| 482 | TTYH1 |  |  | 9872 | -1.033 | -0.0668 | Yes |
| 483 | GPM6B |  |  | 9873 | -1.091 | -0.0546 | Yes |
| 484 | PTN |  |  | 9874 | -1.107 | -0.0423 | Yes |
| 485 | GFAP |  |  | 9877 | -1.174 | -0.0295 | Yes |
| 486 | BCAN |  |  | 9878 | -1.175 | -0.0164 | Yes |
| 487 | SCRG1 |  |  | 9879 | -1.473 | 0.0000 | Yes |
Table: GSEA details [plain text format]

  

Fig 2: RICHARDS\_NOWAKOWSKI\_SCIENCE\_2017\_ASTROCYTE\_UPREG: Random ES distribution      
 Gene set null distribution of ES for **RICHARDS\_NOWAKOWSKI\_SCIENCE\_2017\_ASTROCYTE\_UPREG**
